# Chemical Modifications of Normal and Waxy Potato Starches Affect Functional Properties of Aerogels

**DOI:** 10.3390/gels8110720

**Published:** 2022-11-08

**Authors:** Joanna Le Thanh-Blicharz, Jacek Lewandowicz, Zuzanna Małyszek, Hanna Maria Baranowska, Przemysław Łukasz Kowalczewski

**Affiliations:** 1 Department of Food Concentrates and Starch Products, Prof. Wacław Dąbrowski Institute of Agriculture and Food Biotechnology—State Research Institute, 40 Starołęcka St., 61-361 Poznań, Poland; 2 Institute of Logistics, Poznan University of Technology, 2 Jacka Rychlewskiego St., 60-965 Poznań, Poland; 3 Department of Physics and Biophysics, Faculty of Food Science and Nutrition, Poznań University of Life Sciences, 38/42 Wojska Polskiego St., 60-637 Poznań, Poland; 4 Department of Food Technology of Plant Origin, Poznań University of Life Sciences, 31 Wojska Polskiego St., 60-624 Poznań, Poland

**Keywords:** ^1^H NMR, relaxation times, rheological properties, chemically modified starch, quality, emulsion

## Abstract

Aerogels are of increasing interest because of their exceptionally large surface area, porous structure, and low weight. Despite the significant increase in interest in the subject of starch-based aerogels, the number of detailed studies is rather scarce, which is especially evident in the case of chemically modified derivatives. Therefore, the study aims to evaluate the physicochemical properties of aerogels from chemically modified potato starch preparations (E 1422 and E 1450) obtained both from normal and waxy starches. Aerogels were prepared through the retrogradation of starch pastes followed by the gradual replacement of water with ethyl alcohol. The obtained preparations were characterized in terms of their bulk density, oil-binding capacity, as well as the texture and rheological properties of the formed pastes. Moreover, their usefulness was evaluated in an emulsion system employing rheological and low-field NMR methods. The obtained aerogels were characterized by a lower bulk density of 0.18–0.59 g/cm^3^ and 5.4–6.6 times higher oil-binding capacity compared to native potato starch. The chemical modification of starch helped to further alter the functional properties of the obtained aerogels, making them more effective oil binders, emulsifiers, and stabilizers (increasing the stability from 55 to 90%), which was especially evident for E 1450 preparation. Amylose content improved the aerogel properties, as waxy preparations were characterized by worse functional properties with the only exception of improved thickening ability. The most beneficial properties for the preparation of emulsions were observed for the aerogel obtained based on E 1450 normal potato starch.

## 1. Introduction

Among naturally occurring polymers, starch is characterized by the greatest diversity of physicochemical properties, and is significantly influenced by the biodiversity of the raw materials from which it is obtained [1,2,3,4,5]. Although, for economic reasons, most of the starch produced in the world is derived from corn, wheat, and potatoes, in small amounts (less than 5% in total), it is obtained from other plant materials. The functional properties of starch for use in food production are determined by the rheological properties of its pastes [6,7,8]. The rheological properties of starch pastes are determined by both the ratio of amylose to amylopectin and the molecular structure of both carbohydrates [9,10]. A significant impact on the development of molecular structure and proportion of these starch constituents is caused by its botanical origin as well as a variety of plants from which starch is derived [11,12]. The use of starch for the production of aerogels has been widely studied in recent years [13]. One of the advantages over inorganic aerogels is the possibility of modifying the hydroxyl groups of polysaccharide chains, which in turn allows modifying the properties of bio-aerogels.

Aerogels are of increasing interest because of their exceptionally large surface area, porous structure, and low weight. They have been studied in several applications such as heat insulators, support materials, fillers, and filters. Depending on the parameters used for their production, numerous materials can be created with a wide range of possibilities and properties [14]. The most popular are silica aerogels, mainly used as a component of modern thermal insulation. However, the emphasis is on using natural, renewable, biodegradable, and biocompatible resources to create aerogels, e.g., with starch [15]. Polysaccharide aerogels are gaining wider interest due to their open-pore nanostructure, high surface area, biodegradability, and biocompatibility [16]. The most popular way to create starch aerogels is to dissolve starch in water and retrograde it (forming a hydrogel), gradually replace the water with alcohol, and dry. The drying step of wet polysaccharide gel is crucial to maintain the integrity of the original three-dimensional structure. This step can be accomplished through means of air-, freeze-, or supercritical fluid drying [17,18].

The interest in starch as a raw material for the production of aerogels was initiated by Glenn and Irving in 1995 [19]. The usefulness of starch as a raw material for aerogel production is mainly related to the fact that it is a biodegradable, inexpensive, and abundant food-grade raw material [20]. Despite the significant increase in interest in this subject, information about starch-based aerogels is relatively general. Most studies have been published using corn or wheat starch as a raw material. The exception is the work of Druel et al. [21] in which the effect of the botanical origin of starch and the method of processing on the properties of aerogels was described. However, there is a lack of information of the effect of chemical modification of starch on the properties of aerogels obtained on their basis. In addition, Zhu, in his review of the methods of production and potential applications of starch aerogels, indicated the importance of establishing the effect of starch modification on the properties of obtained aerogels [13].

At the current stage, the applicability of starch aerogels is mostly experimental and potential fields of application are still to be discovered [13]. Aerogels are known for oil-binding properties, with a predominance of the physical nature of the uptake process [22]. The modification of starch can presumably further enhance this property by the incorporation of hydrophobic moieties in the process of starch esterification with acetic or octenyl succinic anhydrite (OSA) [23]. It was also proven that aerogels derived from waxy starch varieties have weaker water–biopolymer interactions when compared to their normal counterparts [24] which can further enhance oil-binding properties. Acetylated distarch adipate (E 1422) and OSA starch (E 1450) are of particular interest due to their common use as emulsifiers in food technology. Therefore, the aim of the study was to evaluate the physicochemical properties including the oil-binding capacity of aerogels from chemically modified starches (E 1422 and E 1450) obtained from normal and waxy potato starch.

## 2. Results and Discussion

### 2.1. Aerogels Characteristics

**Figure 1:**
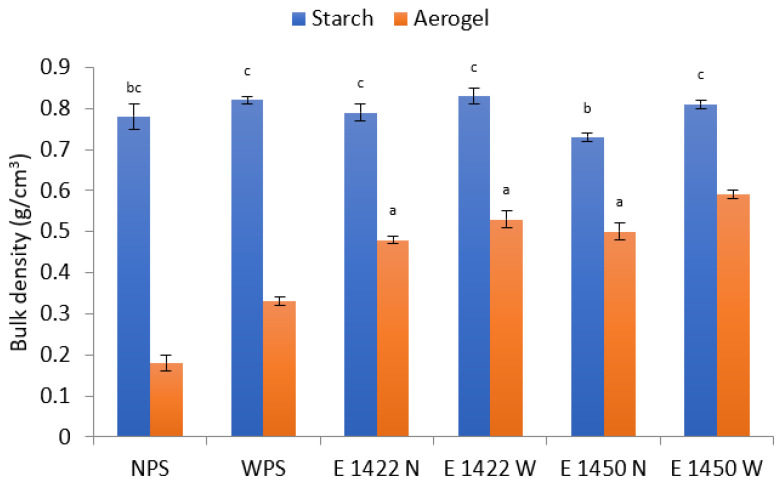
Bulk density of aerogels and the corresponding starch raw materials. Mean value ± standard deviation. Samples denoted with the same letter do not differ significantly *p* > 0.05.

Bulk density is one of the basic parameters characterizing aerogels. Its typical value is usually reported around 0.2 g/cm^3^, and for bio aerogels, even lower-g/cm^3^. The properties of aerogels based on starch depend on the botanical origin, especially the amylose content [21]. The method of aerogel formation reveals also a significant effect [25]. The values presented for native starch aerogels (0.18–0.33 g/cm^3^) in Figure 1 are consistent with the literature data, especially since the waxy-starch-based aerogel had a higher bulk density. Similar observations on the effect of amylose on bulk density were reported for corn starch in which the high-amylase variety had a bulk density of 0.10 g/cm^3^, whereas the normal one was 0.24 g/cm^3^ [19]. The chemical modification resulted in an increase in the value of the bulk density of aerogels, but in all cases, the products based on waxy starch showed a higher value of this parameter than those based on normal starch. This is a potentially disadvantageous phenomenon as a lower bulk density is accompanied by a larger specific surface area [13,15,17,21,25].

**Figure 2:**
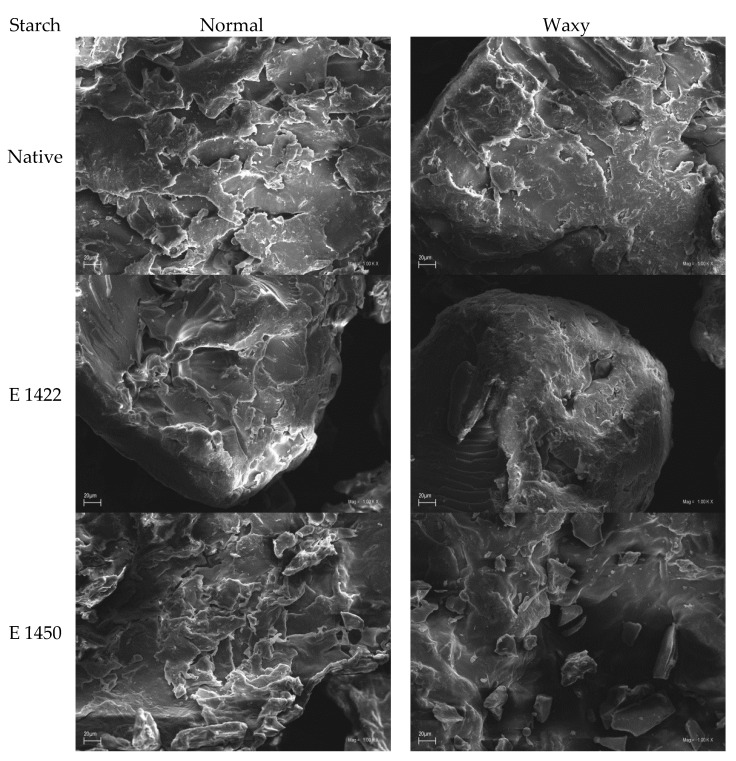
Scanning electron microphotographs of potato starch aerogels.

Native potato starch, regardless of its variety, is characterized by the similar shape of granules, which are round or oval spheres with a smooth surface. Moreover, they are relatively large in size with a median of approximately 35 μm and a broad granule size distribution with extreme deciles reaching as low as 17 μm and as high as 59 μm [26]. As a result of the aerogel preparation procedure, the starch granular form was lost (Figure 2), which was expected as the initial stage of this process consisted of the preparation of starch paste. The structure of the obtained product was much larger and characterized by a rough surface. The roughness of the surface was caused by the presence of numerous asymmetrical cracks and caves that were roughly between 10 and 30 μm in size. The chemical modification of starch used for the production of aerogel led to a decrease in its surface roughness, but the formed caves were larger. This phenomenon partially explains the higher bulk density of those preparations, that could have been caused by the interlocking of smaller aerogel parts inside large caves. A similar pattern was observed between normal and waxy preparations. The corresponding aerogels obtained based on different potato varieties were quite similar, but with a slightly smoother surface. Consequently, this resulted in the higher bulk density of aerogels derived from waxy preparations.

**Figure 3:**
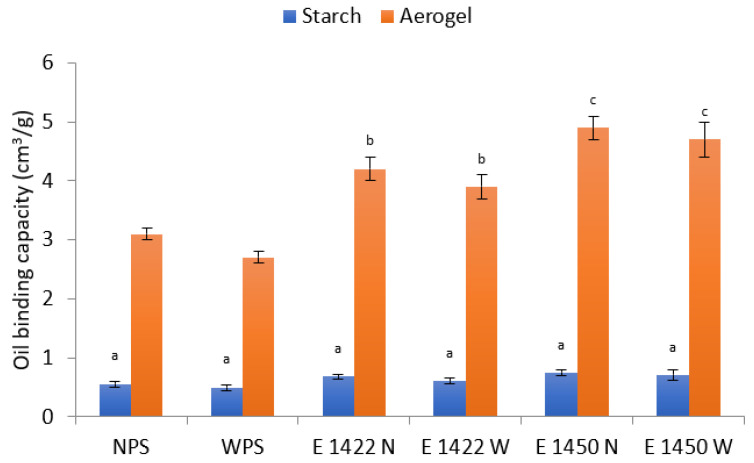
Oil-binding capacity of aerogels and the corresponding starch raw materials. Mean value ± standard deviation. Samples denoted with the same letter do not differ significantly *p* > 0.05.

Aerogels have a highly developed surface so it can be assumed that they are able to adsorb much more oil than native starches, as confirmed in our study (Figure 3). As a result of physical modification that led to the formation of aerogel, the oil-binding capacity increased between 5.4 and 6.6 times. In all cases, aerogels based on waxy starches showed a worse oil adsorption capacity than those based on their normal counterparts. Surprisingly, aerogels based on both chemically modified starches revealed higher oil sorption capacity than the preparations obtained from native starches, in spite of the fact that their higher bulk density would suggest the opposite phenomenon. It is probably related to the type of chemical modification. Acetylated distarch adipate E 1422 is the most popular starch used as an emulsion stabilizer [27]. OSA starch E1450 was designed mainly as an emulsifier [27,28] and is also referred to as hydrophobic starch. It should be noted that the difference in oil adsorption capacity between the E1422 and E 1450 starch-based aerogels did not turn out to be as great as expected between the stabilizer and the emulsifier. The comment for this observation can be found in the study by Prochaska et al. [23] in which only a slight difference in the surface activity of E 1422 and E 1450 starches was observed. Nevertheless, two-way ANOVA, performed between chemically modified aerogel samples only, indicated that the type of chemical modification plays a significant (*p* = 0.0423) role in oil-binding effectiveness, whereas the effect of starch variety is insignificant (*p* = 0.1257).

The rheological properties of starch are crucial for its functionality in food products [29,30]. As starch pastes are pseudoplastic and thixotropic non-Newtonian liquids, their flow characteristics are commonly described using the Ostwald de Waele model. The equation used was characterized by a very good fit to the experimental data (flow curves are included in the Supplementary Materials, Figure S1) with values of R^2^ exceeding 0.95. As can be seen in Table 1, the increase in consistency index was accompanied, as expected, by a decrease in the flow behavior index, which proved more non-Newtonian flow characteristics. Moreover, the increase in consistency index was accompanied by an increase in the value of the area of the hysteresis loop of thixotropy. This phenomenon was also observed for native [30] as well as chemically modified potato starch pastes [31]. The most spectacular observation is the higher consistency index values of aerogels based on waxy starch than their equivalents made of normal starch, as it is known that native waxy varieties of starch reveal lower consistency indexes and lower thixotropy than normal starches [30,32]. Native normal potato starch paste (5%) was reported to have a consistency index of 31.9 Pa·s^n^ and flow behavior index of 0.468, whereas the waxy one had 10.9 Pa·s^n^ and 0.495, respectively [30]. This indicates that, as a result of physical modification in order to obtain aerogel, starch viscosity decreases similarly as in the case of the most common physical modification—pregelatinization [33].

The chemical modification of starch used for aerogel production resulted in an increase in consistency indexes and thixotropy, especially in the case of OSA preparation. Extremely high values of the consistency index accompanied by low values of the flow index for the OSA starch are most likely due to the strong gelling phenomenon of this sample. The observed characteristics are especially atypical, as starch crosslinking is the standard method for increasing the viscosity of starch pastes, while modification with octenylsuccinic anhydride is carried out primarily to impart emulsifying properties to the starch [27,28,31,34].

Texture is a multiparameter sensory property of a food product which is perceived by various human senses. The perception is mostly related to a product’s macro-, micro- and molecular structure [35]. Food products are classified into categories in which texture is of critical, major, or minor importance [36]. The knowledge of the starch texture properties is extremely important from the perspective of its use in the food industry, as their sensory acceptability is of key importance. The results of the universal texture profile (TPA) parameters are presented in Table 2.

The highest values of hardness, adhesiveness, and gumminess were found for aerogels based on OSA starch (E 1450). These results are consistent with rheological data, as E 1450 preparations formed highly viscous and thixotropic pastes (Table 1). Next in terms of texture-promoting properties were aerogels based on E 1422 starch, and the lowest texture values were recorded for starches which were not subjected to chemical modification. This phenomenon is in contradiction to chemically modified starches as the esterification of starch usually results in a minor decrease in texture-promoting properties, which is related to the stabilization of rheological properties [31]. However, this observation is directly linked to the decrease in viscosity caused by the physical modification process. It was also noticed that in each of the investigated cases, the preparations based on starch from waxy varieties had slightly lower values of texture parameters than the preparations obtained from conventional types of starch. This latter observation is in agreement with the literature data reported for waxy starches [30,32]. The same order followed absolute values of adhesion, whereas the springiness of all the samples did not differ significantly between normal and their waxy counterparts.

### 2.2. Emulsions Characteristics

The stability of model emulsions which, apart from oil and water, contain only starch as the emulsifying and stabilizing agent, enables the clear assessment of the functionality of studied preparations in food production. As can be seen in Table 3, aerogels based on native starches exhibited the worst emulsifying–stabilizing effect in model emulsions, achieving an emulsion stability of 65% and 55% for the normal and waxy variety, respectively. However, it should be emphasized that physical modification taking place during the procedure of production of aerogels made them useful as an emulsifying agent, as native starches do not possess this ability. Both types of chemical modification had a positive effect on the emulsion stability, which was especially evident in the case of OSA starch. The reason behind that phenomenon presumably can be explained by differences in the surface activity of both modified starches [23]. Moreover, Saari et al. [37] indicated that ethanol-precipitated OSA starch produces more stable emulsions when compared to conventionally dissolved starch. In all investigated cases, waxy-starch-based aerogels were characterized by poorer emulsion stability, but the observed differences were statistically insignificant. This phenomenon was also observed in mayonnaise thickened with native potato starch, in which the normal variety performed worse [26].

The rheological characteristics of emulsions (Table 3) did not follow the same pattern observed for starch pastes. The employed Ostwald de Waele model was very well-fitted to the experimental data with values of R^2^ exceeding 0.95. One of the factors influencing that phenomenon was the extremely high shear forces applied during emulsion formation. Nevertheless, the rheological studies confirmed the observations on the stability of model emulsions. Viscosity, expressed by the consistency index value, obtained with the use of an aerogel based on normal potato starch, was higher than that observed for waxy counterparts. This may suggest a higher stability of those emulsions, as high viscosity in one of the factors slows the emulsion aging processes [38]. However, the higher values of thixotropy which is a measure of rheological instability [3] may suggest the poorer stability when shear forces are applied. These observations indicate that deterioration in the quality of an emulsion prepared with normal starches will likely take place during processing (due to thixotropic properties), while it will be more stable during storage (stabilization via high viscosity). Accordingly, the lowest stability was observed for the emulsion prepared with unmodified waxy-potato-starch aerogel, which had the lowest consistency index among the investigated samples. The chemical modification of starch increased the consistency index of the formed emulsion based on both waxy aerogel preparations, thus indicating a favorable effect of chemical modification on the stabilization of the emulsions via the viscosity mechanism.

Low-field magnetic resonance studies provide important information on the quality of emulsions due to the strong correlation between their stability and rheological properties [2,39,40]. With the use of this analytical method, it is possible to study proton relaxation phenomena occurring as a consequence of adsorption of the electromagnetic field energy. The proton dynamics of water are most often studied, but it is also possible for fats. The proton relaxation processes occur as a result of the spin energy transfer to the surrounding environment, which is described by the spin–lattice relaxation time T_1_ and by the energy exchange described by the spin–spin relaxation time T_2_. In biopolymer solutions, T_1_ is always higher than T_2_ [41]. In emulsions, two components of the relaxation times are observed (Table 4). This relates to two proton fractions relaxing at different rates in the system, and that the chemical exchange between these proton fractions is much slower than the relaxation time. The long components (T_12_ and T_22_) reflect the relaxation processes of the proton fraction associated mainly with the starch paste, while the short components (T_11_ and T_21_) relate to the oil fraction of the emulsion. In pure oil, the values of the spin–lattice and spin–spin relaxation times are, respectively, T_1_ = 102.8 ± 0.8 ms and T_2_ = 88.4 ± 1.1 ms. In emulsions, the values of the short components are significantly lower than the values of T_1_ and T_2_ in pure oil [39,42,43]. As can be seen in Table 4, the type of starch in the water phase influenced the values of the long T_12_ and T_22_ components of the relaxation times that have been reported in the literature [39,44,45,46]. It is an effect of the entrapment of water molecules by the colloidal lattice [42]. It was higher for the aerogel obtained from the normal native potato starch than from the waxy variety (Table 4). The chemical modification had the opposite effect. Aerogels based on the waxy starch immobilized protons of the water phase more effectively than the normal one. Regarding the oil phase of the formed emulsions, the short component values were significantly lower than the values of T_1_ and T_2_ in oil. The degree of shortening of the relaxation times depended on the type of starch. The effect of the raw material used for obtaining the aerogel on T_11_ time values was multidirectional. However, in the case of the T_21_ time, the chemical modification of the raw material applied to obtain aerogels made the product more efficient in the immobilization of protons of the oil phase. This can be explained by the presence of hydrophobic sequences in the structure of ester substituents, which increases the affinity of starch for the oil phase. This is also the reason for the surface activity exhibited by E 1422 and especially E 1450 starch preparations [23].

### 2.3. Statistical Analysis

**Figure 4:**
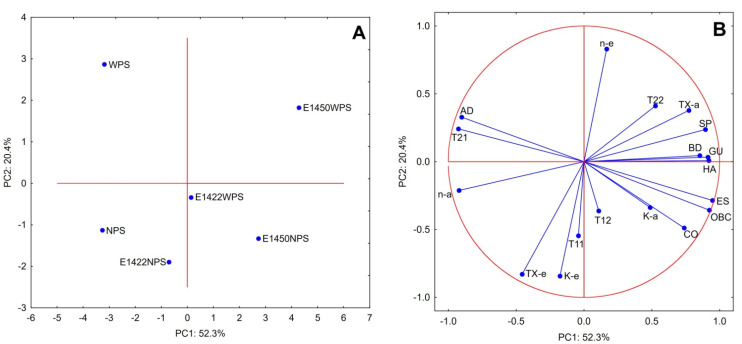
PCA score (**A**); loadings plot (**B**); physicochemical properties of aerogels, as well as their pastes and emulsions. Explanatory notes: BD—bulk density; OBC—oil-binding capacity, K-a—consistency index (paste); n-a—flow behavior index (paste); TX-a—thixotropy (paste); K-e—consistency index (emulsion); n-e—flow behavior index (emulsion); TX-e—thixotropy (emulsion); HA—hardness; AD—adhesiveness; CO—cohesiveness; SP—springiness; GU—gumminess; ES—emulsion stability; T_11_—short component of spin–lattice relaxation time; T_12_—long component of spin–lattice relaxation time; T_21_—short component of spin–spin relaxation time; T_22_—long component of spin–spin relaxation time.

In order to systematize the obtained data on the properties of starch aerogels and emulsions formed by them, principal component analysis was performed. The presented PCA plots (Figure 4) explain 72.7% of the total variance between the investigated samples. The first component (PC1) separates samples by the type of chemical modification employed, and the attributed values increase in the following order: native, E 1422, and 1450. The PC2 differentiates samples based on the starch variety and normal ones are characterized by lower values than waxy ones. The PCA score plot highlights the unique properties of waxy variety as well as OSA preparations.

The PCA loadings plot pointed to a few noticeable corrections between the investigated parameters. The stability of the obtained emulsion was positively correlated with the oil-binding capacity of the aerogel used for its production. The spin–lattice relaxation rate associated with oil fraction of the emulsion (T_11_) was positively correlated with the consistency index. Both of these parameters were also negatively correlated with the flow behavior index, whereas the consistency index of the aerogel paste was correlated with cohesiveness. Breakdown and TPA parameters including hardness and gumminess were positively correlated with each other as well as negatively with adhesiveness. The observations regarding correlations between rheological and texture parameters are in accordance with PCA data obtained for chemically modified starch pastes [31]. The remaining potential correlations should be considered as random.

## 3. Conclusions

The physical modification of starch by the retrogradation of starch pastes followed by the gradual replacement of water with ethyl alcohol is an effective method for the preparation of aerogels from native potato starch. The obtained preparations are characterized by several times lower bulk density and higher oil-binding capacity. Chemical modification can further alter the functional properties of the obtained aerogels. The introduction of relatively hydrophobic moieties into the starch macromolecule, i.e., E 1422 or E 1450, leads to an increase in bulk density which, surprisingly, is accompanied by an increase in oil-binding capacity. Consequently, chemically modified aerogels are effective emulsifiers and stabilizers, which is especially evident in the case of OSA starch. Lastly, the amylose content of starch plays a significant role in shaping the functional properties of potato starch aerogels. Preparations derived from the waxy variety are characterized by higher bulk density and lower oil-binding capacity compared to their normal counterparts. Moreover, waxy-starch aerogels form pastes of high viscosity, and tend to form fewer centrifugal stable emulsions. Therefore, the aerogel obtained from E 1450 derivative based on normal potato starch should be recommended for oil-binding and emulsion-forming applications. Further research should consider the potential effect of the degree of substitution with OSA anhydrite on the observed properties. Additionally, studies on aerogels produced from starch preparations isolated from high amylose plant varieties may provide more efficient products. Finally, research on performance in different food product matrices needs to be considered.

## 4. Materials and Methods

### 4.1. Aerogels Making

The study used native potato starch (PPZ Trzemeszno, Trzemeszno, Poland) and waxy potato starch (“Eliane 100”, Avebe, Veendam, The Netherlands) as well as acetylated distarch adipate (E 1422) prepared according to the procedure described by Luo et al. [47] and sodium octenyl succinate starch (E 1450) prepared according to the procedure described by Jeon et al. [48] obtained from native and waxy starches.

**Figure 5:**
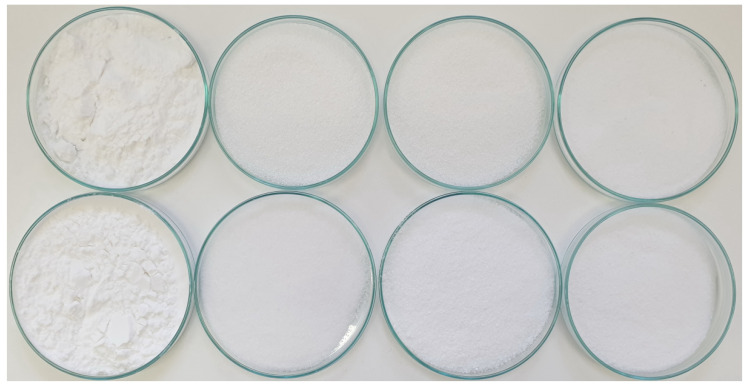
Photograph of raw material and obtained aerogel samples (from left: native starch, aerogel, E 1422 aerogel, and E1450 aerogel; from top: native and waxy variety, respectively).

Aerogels were prepared using a method consisting of the successive displacement of water from the gel network structure by ethanol [25] in accordance with the method described in detail by the team of Le Thanh-Blicharz et al. [24]. In brief, 7% starch pastes were frozen at −15 °C, thawed, and washed with ethyl alcohol. This procedure was followed thrice and afterwards, the aerogels were dried at 50 °C to a constant weight. The obtained samples, presented in Figure 5, were denoted as follows: “NPS” aerogels from normal potato starch and “WPS” from waxy potato starch. The modified starch aerogel samples were coded according to the International Numbering System and further denoted a N or W for the normal and waxy variety, respectively: “E 1422N”, “E 1422W”, “E 1450N”, and “E 1450W” from starch E1422 and E1450.

### 4.2. Preparation of Aerogel Pastes

Starch aerogel was suspended in deionized water (5 g/100 mL) and placed in a boiling-water bath for 15 min under continuous stirring (R50D, CAT) at 150 rpm. The prepared aerogel starch pastes were allowed to set at 22 ± 2 °C for 1 h prior to further analysis. All further investigations were made on freshly prepared pastes unless otherwise stated.

### 4.3. Preparation of Emulsions

The emulsions were prepared by mixing rapeseed oil and deionized water in a ratio of 1:3. The starch concentration was 5%. To prepare the emulsion, aerogel powders were dispersed in rapeseed oil; afterwards, the water was added and the sample was immediately homogenized using a Silent Crusher homogenizer (Heidolph, Schwabach, Germany) with a rotation speed of 21,000 rpm for 3 min.

### 4.4. Analytical Methods

#### 4.4.1. Bulk Density

The bulk density of the aerogels was determined using the method described by Kaur, Kaushala, and Sandhu [49]. In brief, 100 g of each aerogel was gently poured into the measuring cylinder with a volume of 100 cm^3^ and then the bottom of the measuring cylinder was gently tapped ten times on the laboratory table. Bulk density was calculated as the weight of the aerogel divided by the volume of the sample.

#### 4.4.2. Microstructure

The scanning electron microphotographs were taken with the assistance of an Evo 40 series microscope (Carl Zeiss, Jena, Germany) operating at 17 kV accelerating voltage. Samples were coated with gold using an SCD 050 sputter coater (Oerlikon Balzers, Lihtenstein).

#### 4.4.3. Oil-Binding Capacity

The oil-binding capacity (OBC) was determined using the method described by Jeżowski et al. [50] with a slight modification. 10 g of starch and 100 mL of rapeseed oil was shaken for 3 min using a laboratory shaker; then, the starch was drained and dried. The oil absorption is expressed as the volume of oil (mL) bound by 1 g of aerogel.

#### 4.4.4. Rheological Properties

The rheological properties of starch pastes and emulsions were determined using a RotoVisco1 rheometer (Haake Technik GmbH, Vreden, Germany) equipped with Z20 DIN coaxial measurement geometry. The samples were allowed to rest in a measuring cylinder for 5 min at 20 °C. The flow curves were determined at a controlled shear rate in the 1-600-1 s^−1^ range for a time of 60 s for both upwards and downwards curves. The obtained data were fitted to the Ostwald de Waele model using RheoWin v. 3.61 (Haake Technik GmbH, Vreden, Germany) software:

(1) $$\tau =K\cdot \;{\dot{\gamma }}^{n}$$

where τ—shear stress (Pa), *K*—consistency index (Pa·s^n^), and $\dot{\gamma }$—shear rate.

#### 4.4.5. Texture Profile Analysis

The universal texture profile (TPA) of the aerogel pastes was determined with a TA.XT2 texturometer, Stable Micro Systems (Godalming, UK). The “double bite test” was performed with a 35 mm-diameter aluminum cylindrical probe and at a distance of 20 mm and test speed of 0.5 mm/s. The following TPA parameters were determined: Hardness (N), adhesiveness (N·s), cohesiveness, springiness, and gumminess (N).

#### 4.4.6. Emulsion Stability

Emulsion stability was determined according to the procedure of Acton and Saffle [51]. The emulsion samples were placed in centrifuge tubes and thermostated at 37 °C for 24 h. Subsequently, the samples were centrifuged at 2500× *g* during 10 min. Emulsion stability (ES) in % was calculated from the following formula

(2) $$ES=\frac{V_{e}-V_{o}}{V_{e}}\;\cdot 100\%$$

where *V_e_*—initial volume of emulsion (cm^3^) and *V_o_*—volume of separated oil (cm^3^).

#### 4.4.7. LF NMR of Emulsions

Low-field NMR measurements were taken with the assistance of a PS20 pulse spectrometer operating at 20 MHz (Ellab, Poznań, Poland). To measure the spin–lattice relaxation time T_1_, an inversion recovery pulse sequence (π-τ-π/2) was used [39,40]. The distances τ between RF pulses varied from 40 to 1100 ms. A total of 119 points were collected in each of 32 free induction decay signals. The repetition time for each measurement was 15 s. The T_1_ relaxation times were calculated using CracSpin software [52], in which the Marquardt method enables minimization by adjusting to the multi-exponential course of the magnetization regrowth curve. The increase in the longitudinal magnetization component (*M_z_*) is described by the formula:

(3) $$M_{z}\left(\tau \right)=M_{0}\left(1-2\overset{n}{\underset{i=1}{\sum }}p_{i}exp\left(\frac{-\tau }{T_{1i}}\right)\right)$$

where *M_z_*(*t*) is the actual magnetization value, *M*_0_ is the equilibrium magnetization value, and *p_i_* is the fractions of protons relaxing with T_1*i*_ relaxation times.

A bi-exponential regrowth of magnetization was noted.

In order to measure spin–spin relaxation times (T_2_), the CPMG pulse train (π/2-*t*/2(π-*t*)*_n_*) was used [39]. The distances between the pulses of π(*t*) were 2 ms. The repetition time between the sequences was 15 s. Each sequence consisted of 100 spin echoes. The decay of the spin–echo amplitudes (*M_x_*_,*y*_) is described by the formula:

(4) $$M_{x,y}\left(\tau \right)=M_{0}\;\overset{n}{\underset{i=1}{\sum }}p_{i}exp\left(\frac{-\tau }{T_{2i}}\right)$$

where *M_x_*_,*y*_(*t*) is the actual echo amplitude; *M*_0_ is the equilibrium amplitude; and *p_i_* is the fraction of protons relaxing with the T_2*i*_ spin–spin time.

For the calculations of the T_2_ relaxation times, the TableCurve v. 5.1 (Inpixon HQ, Palo Alto, CA, USA) program was used and the least squares method was used to fit the decay of the spin echoes’ amplitudes to the above formula.

### 4.5. Statistical Analysis

The aerogels were synthetized three times and each synthetized material was analyzed three times, unless otherwise stated. Statistical analysis of the data was performed using the Statistica v. 13.3 software (Dell Software Inc., Round Rock, TX, USA). The data were studied using one-way analysis of variance. Tukey’s honest significant difference post hoc test was used to determine statistically homogeneous subsets at α = 0.05. Principal component analysis (PCA) was performed based on a correlation matrix using selected data obtained in the analyses.

## Figures and Tables

**Table 1:** Parameters of the Ostwald de Waele equation for pastes of aerogels of concentration of 5%.

Sample	K (Pa·s^n^)	*n* (−)	Thixotropy (Pa·s^−1^)
NPS	7.04 ± 0.25 ^a^	0.659 ± 0.009 ^a^	744 ± 492
WPS	9.91 ± 0.19 ^a^	0.635 ± 0.005 ^a^	19,020 ± 1420 ^a^
E 1422 N	12.15 ± 0.60 ^a^	0.578 ± 0.014	26,290 ± 2820 ^b^
E 1422 W	26.62 ± 0.51 ^b^	0.359 ± 0.006	27,470 ± 1947 ^b^
E 1450 N	30.81 ± 1.93 ^b^	0.407 ± 0.031	19,870 ± 2573 ^a^
E 1450 W	112.4 ± 4.68	0.157 ± 0.012	47,770 ± 2793

Mean value ± standard deviation. Samples denoted with the same letter do not differ significantly *p* > 0.05.

**Table 2:** The texture of the pastes of aerogels of concentration of 5%.

Sample	Hardness (N)	Adhesion (N·s)	Cohesiveness (−)	Springiness (−)	Gumminess (−)
NPS	0.39 ± 0.02 ^a^	−0.07 ± 0.01 ^a^	0.79 ± 0.02 ^b^	1.00 ± 0.00 ^a^	0.31 ± 0.02 ^ab^
WPS	0.37 ± 0.01 ^a^	0.00 ± 0.00 ^a^	0.72 ± 0.01 ^a^	1.00 ± 0.00 ^a^	0.27 ± 0.01 ^a^
E 1422 N	0.52 ± 0.04 ^a^	−1.80 ± 0.27 ^bc^	0.79 ± 0.02 ^b^	1.00 ± 0.00 ^a^	0.41 ± 0.02 ^c^
E 1422 W	0.49 ± 0.04 ^a^	−1.30 ± 0.23 ^b^	0.77 ± 0.01 ^ab^	1.00 ± 0.00 ^a^	0.38 ± 0.02 ^bc^
E 1450 N	1.38 ± 0.11 ^b^	−2.30 ± 0.32 ^c^	0.81 ± 0.03 ^b^	1.05 ± 0.02 ^b^	1.45 ± 0.06 ^d^
E 1450 W	1.32 ± 0.12 ^b^	−2.10 ± 0.25 ^c^	0.82 ± 0.02 ^b^	1.08 ± 0.02 ^b^	1.43 ± 0.05 ^d^

Mean value ± standard deviation. Values marked with the same letter do not differ significantly *p* > 0.05.

**Table 3:** Stability of model emulsions made with aerogels and their rheological parameters.

Sample	Emulsion Stability (%)	K (Pa·s^n^)	*n* (−)	Thixotropy (Pa·s^−1^)
NPS	65.0 ± 2.0	4.51 ± 1.01 ^c^	0.325 ± 0.021 ^a^	4235 ± 1086 ^c^
WPS	55.0 ± 2.9	0.89 ± 0.01 ^a^	0.573 ± 0.007 ^c^	1083 ± 288 ^a^
E 1422 N	75.0 ± 2.9 ^a^	3.64 ± 1.66 ^bc^	0.362 ± 0.082 ^a^	2898 ± 774 ^abc^
E 1422 W	72.5 ± 2.0 ^a^	1.91 ± 0.51 ^ab^	0.415 ± 0.021 ^ab^	2235 ± 886 ^ab^
E 1450 N	90.0 ± 0.0 ^b^	3.04 ± 0.21 ^abc^	0.463 ± 0.013 ^b^	3061 ± 128 ^bc^
E 1450 W	90.0 ± 2.0 ^b^	1.85 ± 0.29 ^ab^	0.478 ± 0.022 ^bc^	2860 ± 219 ^abc^

Mean value ± standard deviation. Samples denoted with the same letter do not differ significantly *p* > 0.05. Flow curves are included in the Supplementary Materials, Figure S2.

**Table 4:** Relaxation times of model emulsions made with aerogels.

Sample	T_11_ (ms)	T_12_ (ms)	T_21_ (ms)	T_22_ (ms)
NPS	76.4 ± 0.2 ^a^	1000.1 ± 3.4 ^bc^	73.4 ± 1.0 ^b^	549.6 ± 2.7
WPS	83.1 ± 1.0 ^b^	981.1 ± 3.3 ^a^	76.0 ± 1.3 ^b^	500.1 ± 2.1
E 1422 N	96.6 ± 1.3	1031.5 ± 3.5	69.2 ±1.0	460.2 ± 1.8
E 1422 W	84.0 ± 0.8 ^b^	1062.5 ± 4.9	63.9 ±1.6 ^a^	511.5 ± 2.8 ^a^
E 1450 N	90.1 ± 2.4	988.6 ± 6.7 ^ab^	61.9 ± 1.9 ^a^	517.7 ± 3.6 ^a^
E 1450 W	74.5 ± 0.9 ^a^	1008.2 ± 3.8 ^c^	62.4 ± 0.8 ^a^	625.2 ± 2.7

Explanatory notes: mean value ± standard deviation. Samples denoted with the same letter do not differ significantly *p* > 0.05.

## Data Availability

All data generated or analyzed during this study are included in this published article.

## Supplementary Materials

The following supporting information can be downloaded at: https://www.mdpi.com/article/10.3390/gels8110720/s1, Figure S1: Flow curves of aerogel paste; Figure S2: Flow curves of aerogel emulsion.

## References

[1] Goswami B., Mahanta D. (2021). Starch and its Derivatives: Properties and Applications. Polysaccharides.

[2] Makowska A., Dwiecki K., Kubiak P., Baranowska H.M., Lewandowicz G. (2022). Polymer-Solvent Interactions in Modified Starches Pastes–Electrokinetic, Dynamic Light Scattering, Rheological and Low Field Nuclear Magnetic Resonance Approach. Polymers.

[3] Krystyjan M., Sikora M., Adamczyk G., Dobosz A., Tomasik P., Berski W., Łukasiewicz M., Izak P. (2016). Thixotropic properties of waxy potato starch depending on the degree of the granules pasting. Carbohydr. Polym..

[4] Sikora M., Dobosz A., Krystyjan M., Adamczyk G., Tomasik P., Berski W., Kutyła-Kupidura E.M. (2017). Thixotropic properties of the normal potato starch—Locust bean gum blends. LWT.

[5] Makowska A., Szwengiel A., Kubiak P., Tomaszewska-Gras J. (2014). Characteristics and structure of starch isolated from triticale. Starch -Stärke.

[6] Sikora M., Adamczyk G., Krystyjan M., Dobosz A., Tomasik P., Berski W., Lukasiewicz M., Izak P. (2015). Thixotropic properties of normal potato starch depending on the degree of the granules pasting. Carbohydr. Polym..

[7] Adamczyk G., Krystyjan M., Dobosz A., Sikora M., Krystjan M., Dobosz A., Sikora M. (2013). Thixotropic properties of starch. Zywnosc Nauka Technol. Jakosc/Food Sci. Technol. Qual..

[8] Adamczyk G., Krystyjan M., Kuźniar P., Kowalczewski P.Ł., Bobel I. (2022). An Insight into Pasting and Rheological Behavior of Potato Starch Pastes and Gels with Whole and Ground Chia Seeds. Gels.

[9] Zhang B., Xiao Y., Wu X., Luo F., Lin Q., Ding Y. (2021). Changes in structural, digestive, and rheological properties of corn, potato, and pea starches as influenced by different ultrasonic treatments. Int. J. Biol. Macromol..

[10] Zhu F. (2021). Structure and physicochemical properties of starch affected by dynamic pressure treatments: A review. Trends Food Sci. Technol..

[11] Blennow A., Engelsen S.B., Munck L., Møller B.L. (2000). Starch molecular structure and phosphorylation investigated by a combined chromatographic and chemometric approach. Carbohydr. Polym..

[12] Hoover R. (2001). Composition, molecular structure, and physicochemical properties of tuber and root starches: A review. Carbohydr. Polym..

[13] Zhu F. (2019). Starch based aerogels: Production, properties and applications. Trends Food Sci. Technol..

[14] Du A., Zhou B., Zhang Z., Shen J. (2013). A Special Material or a New State of Matter: A Review and Reconsideration of the Aerogel. Materials.

[15] Ganesan K., Budtova T., Ratke L., Gurikov P., Baudron V., Preibisch I., Niemeyer P., Smirnova I., Milow B. (2018). Review on the Production of Polysaccharide Aerogel Particles. Materials.

[16] De Marco I., Iannone R., Miranda S., Riemma S. (2018). An environmental study on starch aerogel for drug delivery applications: Effect of plant scale-up. Int. J. Life Cycle Assess..

[17] Santos-Rosales V., Alvarez-Rivera G., Hillgärtner M., Cifuentes A., Itskov M., García-González C.A., Rege A. (2020). Stability Studies of Starch Aerogel Formulations for Biomedical Applications. Biomacromolecules.

[18] Santos-Rosales V., Ardao I., Alvarez-Lorenzo C., Ribeiro N., Oliveira A., García-González C. (2019). Sterile and Dual-Porous Aerogels Scaffolds Obtained through a Multistep Supercritical CO_2_-Based Approach. Molecules.

[19] Glenn G.M., Irving D.W. (1995). Starch-based microcellular foams. Cereal Chem..

[20] Abhari N., Madadlou A., Dini A. (2017). Structure of starch aerogel as affected by crosslinking and feasibility assessment of the aerogel for an anti-fungal volatile release. Food Chem..

[21] Druel L., Bardl R., Vorwerg W., Budtova T. (2017). Starch Aerogels: A Member of the Family of Thermal Superinsulating Materials. Biomacromolecules.

[22] Sharma V., Shahnaz T., Subbiah S., Narayanasamy S. (2020). New Insights into the Remediation of Water Pollutants using Nanobentonite Incorporated Nanocellulose Chitosan Based Aerogel. J. Polym. Environ..

[23] Prochaska K., Kędziora P., Le Thanh J., Lewandowicz G. (2007). Surface activity of commercial food grade modified starches. Colloids Surf. B Biointerfaces.

[24] Le Thanh-Blicharz J., Lewandowicz J., Małyszek Z., Kowalczewski P.Ł., Walkowiak K., Masewicz Ł., Baranowska H.M. (2021). Water Behavior of Aerogels Obtained from Chemically Modified Potato Starches during Hydration. Foods.

[25] Chang P.R., Yu J., Ma X. (2011). Preparation of porous starch and its use as a structure-directing agent for production of porous zinc oxide. Carbohydr. Polym..

[26] Lewandowicz J. (2017). Physicochemical Characteristics and Evaluation of Applicability of Waxy Starches. Ph.D. Thesis.

[27] Ashogbon A.O., Akintayo E.T. (2014). Recent trend in the physical and chemical modification of starches from different botanical sources: A review. Starch -Stärke.

[28] Altuna L., Herrera M.L., Foresti M.L. (2018). Synthesis and characterization of octenyl succinic anhydride modified starches for food applications. A review of recent literature. Food Hydrocoll..

[29] Cornejo-Ramírez Y.I., Martínez-Cruz O., Del Toro-Sánchez C.L., Wong-Corral F.J., Borboa-Flores J., Cinco-Moroyoqui F.J. (2018). The structural characteristics of starches and their functional properties. CyTA -J. Food.

[30] Le Thanh-Blicharz J., Lewandowicz J. (2020). Functionality of Native Starches in Food Systems: Cluster Analysis Grouping of Rheological Properties in Different Product Matrices. Foods.

[31] Lewandowicz J., Le Thanh-Blicharz J., Szwengiel A. (2022). The Effect of Chemical Modification on the Rheological Properties and Structure of Food Grade Modified Starches. Processes.

[32] Liu Y., Chen X., Xu Y., Xu Z., Li H., Sui Z., Corke H. (2021). Gel texture and rheological properties of normal amylose and waxy potato starch blends with rice starches differing in amylose content. Int. J. Food Sci. Technol..

[33] Nakorn K.N., Tongdang T., Sirivongpaisal P. (2009). Crystallinity and Rheological Properties of Pregelatinized Rice Starches Differing in Amylose Content. Starch -Stärke.

[34] Błaszczak W., Lewandowicz G. (2020). Light Microscopy as a Tool to Evaluate the Functionality of Starch in Food. Foods.

[35] Szczesniak A.S. (2002). Texture is a sensory property. Food Qual. Prefer..

[36] Bourne M.C. (2002). Texture, Viscosity, and Food. Food Texture and Viscosity.

[37] Saari H., Wahlgren M., Rayner M., Sjöö M., Matos M. (2019). A comparison of emulsion stability for different OSA-modified waxy maize emulsifiers: Granules, dissolved starch, and non-solvent precipitates. PLoS ONE.

[38] Dickinson E. (2009). Hydrocolloids as emulsifiers and emulsion stabilizers. Food Hydrocoll..

[39] Małyszek Z., Lewandowicz J., Le Thanh-Blicharz J., Walkowiak K., Kowalczewski P.Ł., Baranowska H.M. (2021). Water Behavior of Emulsions Stabilized by Modified Potato Starch. Polymers.

[40] Baranowska H.M., Kowalczewski P.Ł. (2022). Low-Field NMR Analyses of Gels and Starch-Stabilized Emulsions with Modified Potato Starches. Processes.

[41] Kirtil E., Cikrikci S., McCarthy M.J., Oztop M.H. (2017). Recent advances in time domain NMR & MRI sensors and their food applications. Curr. Opin. Food Sci..

[42] Vaclavik V.A., Christian E.W., Vaclavik V.A., Christian E.W. (2008). Water BT—Essentials of Food Science.

[43] Baranowska H.M., Sikora M., Krystyjan M., Tomasik P. (2012). Evaluation of the time-dependent stability of starch–hydrocolloid binary gels involving NMR relaxation time measurements. J. Food Eng..

[44] Baranowska H.M., Rezler R. (2015). Emulsions stabilized using potato starch. Food Sci. Biotechnol..

[45] Baranowska H.M., Rezler R. (2015). Water binding analysis of fat-water emulsions. Food Sci. Biotechnol..

[46] Rezler R., Baranowska H.M. (2013). Rheological and water binding properties of fat-in-water type emulsions stabilized by potato starch. Żywność. Nauk. Technol. Jakość/Food. Sci. Technol. Qual..

[47] Luo F., Huang Q., Fu X., Zhang L., Yu S. (2009). Preparation and characterisation of crosslinked waxy potato starch. Food Chem..

[48] Jeon Y.-S., Lowell A.V., Gross R.A. (1999). Studies of Starch Esterification: Reactions with Alkenylsuccinates in Aqueous Slurry Systems. Starch -Stärke.

[49] Kaur M., Kaushal P., Sandhu K.S. (2013). Studies on physicochemical and pasting properties of Taro (*Colocasia esculenta* L.) flour in comparison with a cereal, tuber and legume flour. J. Food Sci. Technol..

[50] Jeżowski P., Polcyn K., Tomkowiak A., Rybicka I., Radzikowska D. (2020). Technological and antioxidant properties of proteins obtained from waste potato juice. Open Life Sci..

[51] Acton J.C., Saffle R.L. (1970). stability of oil-in-water emulsions. 1. Effects of Surface Tension, Level of Oil, Viscosity and Type of Meat Protein. J. Food Sci..

[52] Weglarz W.P., Haranczyk H. (2000). Two-dimensional analysis of the nuclear relaxation function in the time domain: The program CracSpin. J. Phys. D. Appl. Phys..

