# Supplementary figures and images for: Chemical Modifications of Normal and Waxy Potato Starches Affect Functional Properties of Aerogels
Source: Gels. 2022 Nov 8;8(11):720. doi: 10.3390/gels8110720 (PMC9689880; doi:10.3390/gels8110720)

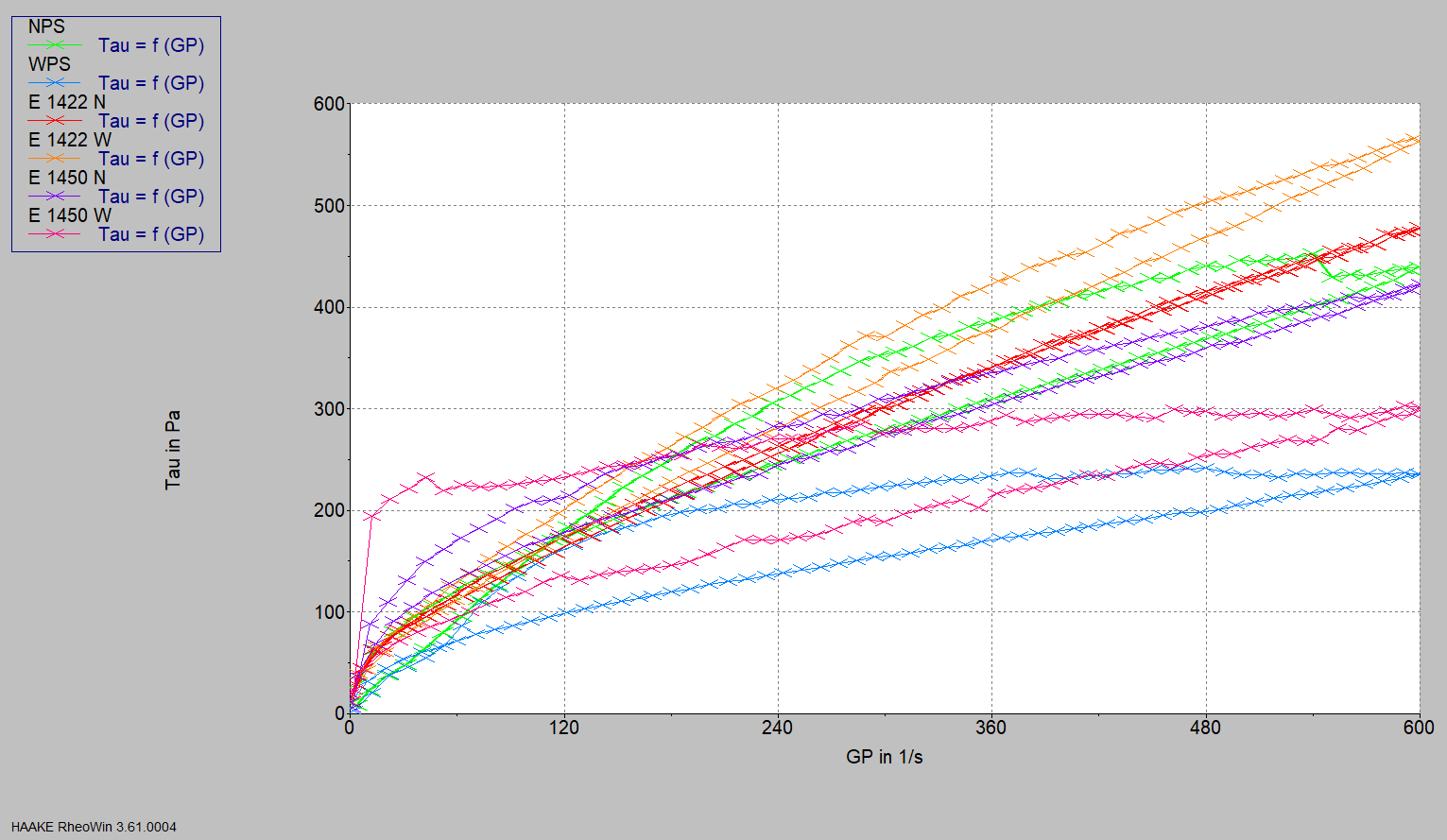

Supplement: Supplementary file 1 [file gels-08-00720-s001.zip › figure_S1_flow_curves_aerogel_paste.png]

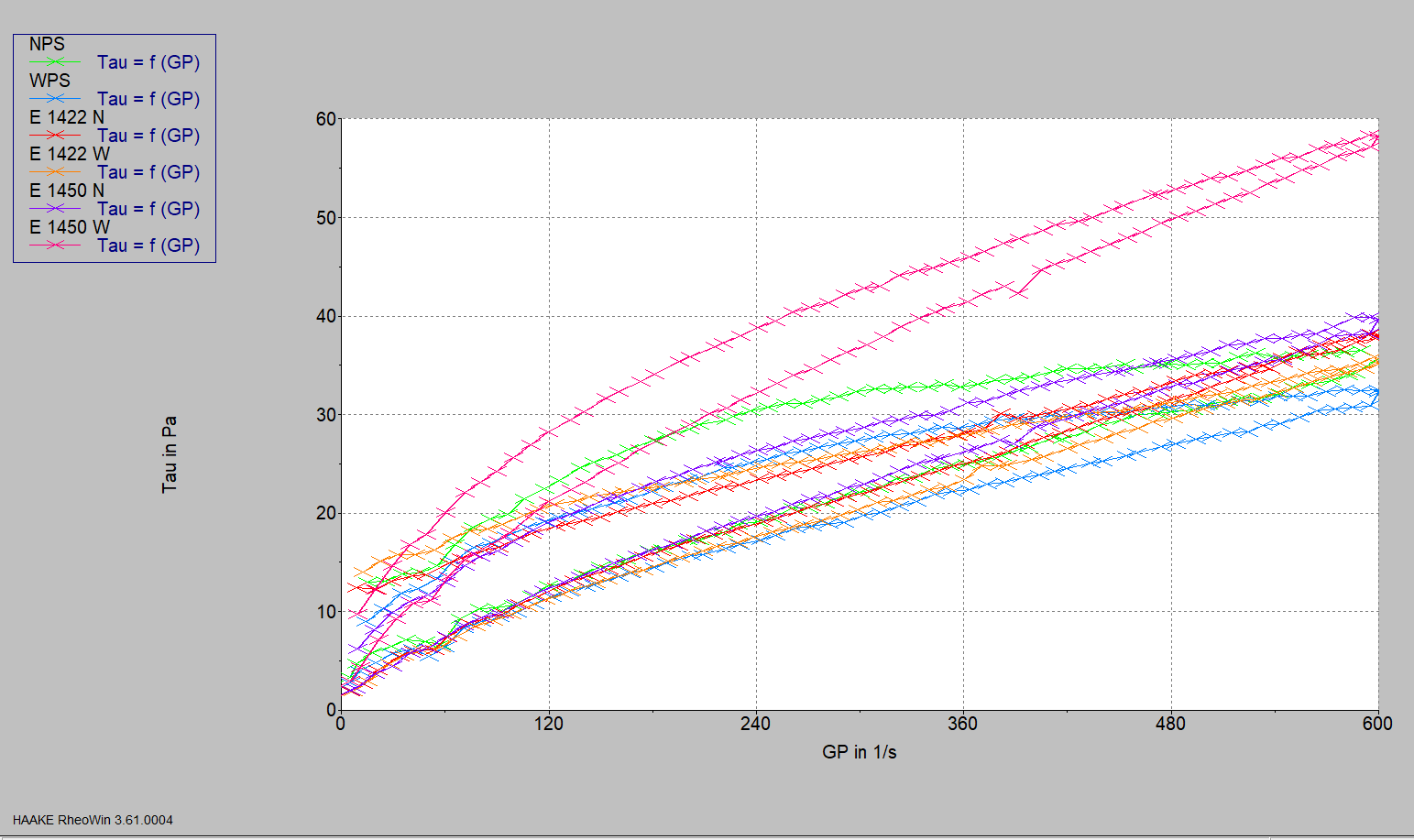

Supplement: Supplementary file 1 [file gels-08-00720-s001.zip › figure_S2_flow_curve_aerogel_emulsion.png]
